# Strengthening national, regional and global health capacity through the WHO Western Pacific Region’s Field Epidemiology Fellowship Programme

**DOI:** 10.5365/wpsar.2021.12.4.844

**Published:** 2021-10-26

**Authors:** Eri Togami, Christopher Lowbridge, Thilaka Chinnayah, Masaya Kato, Munehisa Fukusumi, Jin Gwack, Tamano Matsui, Babatunde Olowokure, Ailan Li

**Affiliations:** a WHO Health Emergencies Programme, World Health Organization Regional Office for the Western Pacific, Manila, Philippines.

## Abstract

**Objective:**

The World Health Organization’s (WHO’s) Field Epidemiology Fellowship Programme in the Western Pacific Region aims to strengthen countries’ capacities for surveillance and risk assessment and build a workforce to tackle public health emergencies. A survey was conducted to assess the on-the-job training experience of the Regional Fellows, evaluate the strengths of the Programme and gain feedback on areas for improvement.

**Methods:**

Between 25 September and 25 October 2018, an online survey was sent to Regional Fellows who had participated in the Programme between July 2006 and September 2018. The survey was shared with WHO country offices in the Western Pacific Region and directly with graduates of the Programme. Responses were recorded electronically and analysed.

**Results:**

A total of 53 former Regional Fellows responded (54% response rate; 53/98). At the time of Programme participation, the Fellows’ median age was 35, 62% (33/53) were female and 72% (38/53) were affiliated with a national or subnational health department. Fellows gained experience in event-based surveillance and risk assessment and worked among a diverse group of professionals in various Member States. Altogether, 77% (41/53) of respondents believed that the Programme had helped them move into a better career position with greater responsibility. Ninety-four percent (50/53) would recommend the Programme to their colleagues.

**Discussion:**

Alumni from the Western Pacific Region’s Field Epidemiology Fellowship Programme perform key health security roles, particularly within governmental systems, and directly contribute to managing health emergencies in their countries, in the Region and globally. The Programme is building a workforce with surge capacity to ensure that public health events in the Region can be addressed. Furthermore, connections developed through the Programme are helping to develop an alumni network, and enhance communications among Member States and between Member States and WHO.

Public health emergencies, such as outbreaks of emerging infectious diseases and natural disasters, pose threats to health security and economies in the World Health Organization’s (WHO’s) Western Pacific Region. (*1*, *2*) Although the occurrence of such events is unpredictable, preparedness, prompt detection and rapid responses can reduce their impacts. In health emergencies, field epidemiologists play vital roles in the detection, verification, risk assessment, response and communication of events at the local, national and regional levels. (*3*) A sufficient pool of competent field epidemiologists is necessary to respond to these events in a timely manner. Field Epidemiology Training Programmes (FETP) and modified Field Epidemiology Training (FET) are implemented by countries, depending on a Member State’s situation, capacity and needs. (*4*)

The WHO Western Pacific Region’s Field Epidemiology Fellowship Programme is an applied epidemiology training programme provided by WHO’s Regional Office for the Western Pacific; for simplicity, participants are referred to throughout this paper as Regional Fellows. The objectives of this Programme are to (i) strengthen countries’ capacities for surveillance and risk assessment, (ii) build a workforce to address public health emergencies, (iii) provide the staff needed for surge capacity responses to public health emergencies, (iv) contribute to and improve WHO’s regional and global event-based surveillance and response systems, and (v) establish a regional network of Programme alumni to facilitate information sharing and collaboration to enhance health security. The Programme achieves these objectives by inviting FETP and FET trainees and graduates in the Region to work with the WHO Health Emergency Information Management and Risk Assessment team in the Health Emergencies Programme, usually for 7 to 9 weeks.

Regional Fellows undergo on-the-job training in a multicultural and diverse work environment, improving their skills by applying an all-hazards approach to event- and indicator-based surveillance; risk assessment; health emergency information management; and responses to emerging infectious diseases, disasters and other unexpected events. Upon completion of the Programme, which may include a field deployment, the Regional Fellows return to their country and are expected to use their new knowledge and skills to contribute towards strengthening national and regional epidemiological and field capacity.

Originally published in 2006, the Asia Pacific Strategy for Emerging Diseases and Public Health Emergencies (APSED III) is the third iteration of a regional framework aimed at implementing, maintaining and advancing the International Health Regulations (IHR) 2005 core capacities in the Asia Pacific. The Western Pacific Region’s Fellowship Programme was established in 2006 to strengthen the capacities of Member States and WHO to rapidly detect and respond to emerging infectious diseases and other acute public health events in the region. This is consistent with developing core capacities under IHR (2005). (*5*)

From 2006 to 2018, more than 130 public health officials, interns and volunteers from 13 Member States participated in the Region’s Fellowship Programme. In 2011, the Programme changed from an individual, mentorship-based experience to a more structured format where Regional Fellows joined a public health intelligence team focused on event-based surveillance, signal verification, risk assessment and response.

Until now, the experiences of the Regional Fellows and their feedback on the Programme had not been systematically evaluated. The objectives of this survey were to capture the on-the-job training experience of the Fellows, evaluate the usefulness and strengths of the Programme as an opportunity for Fellows to develop competencies in surveillance and responding to health emergencies, and gain feedback on areas in which the Programme could be strengthened.

## Methods

### Definitions

For the purposes of this paper, “FET/P” is defined as FET, FETP or equivalent programmes that are implemented in individual countries. FETP is a two-year “learning by doing” training programme for field epidemiology. A modified version of the FETP is the FET, which is usually shorter and adapted to the country’s situation and needs while maintaining on-the-job mentorship and training. We differentiate these FET/Ps from those of the Regional Fellows participating in the WHO Western Pacific Region’s Field Epidemiology Fellowship Programme.

### Survey development and platform

The survey was developed with the online platform KoBoToolbox, a tool developed by the Harvard Humanitarian Initiative. (*6*) KoBoToolbox was selected because it was the most accessible platform for all countries in the Western Pacific Region. The survey consisted of 34 questions, with 5 question types: binary choice, multiple choice, Likert scale, ranking and free text. One question at the beginning of the survey was optional (name).

### Eligibility and survey dissemination

The Western Pacific Region’s database of Regional Fellows was used to select those who had participated in the Programme between July 2006 and September 2018. The URL for the survey was shared via e-mail with WHO country offices in Brunei Darussalam, Cambodia, China, the Lao People's Democratic Republic, Malaysia, Mongolia, the Philippines, Singapore and Viet Nam. Focal points for the WHO Health Emergencies Programme in these countries disseminated the survey to graduates of the Programme in their countries. Alumni from Australia, Japan and the Republic of Korea were contacted directly by the survey team. These two methods were used because not all countries in the Region have a WHO country office. Eligible alumni who did not respond were sent up to two reminder e-mails by the team. The survey link was open for 1 month, from 25 September to 25 October 2018.

A total of 144 fellows were initially identified in the database. Not all participants in the Region’s Fellowship Programme were in FET/Ps at the time of the survey or had previously participated in FET/Ps. Interns and volunteers were not eligible to participate in the survey because their learning needs and career trajectories may differ from those of alumni who were affiliated with Member States’ governmental or other institutions. After removing duplicates, interns, volunteers and Regional Fellows whose active e-mail addresses could not be determined, 98 former Fellows were asked to participate in the survey.

### Analysis

Survey responses were collected via the online platform. After the survey deadline passed, responses were analysed using Microsoft Excel and R statistical software, version 3.1.3. For binary, multiple choice and Likert scale questions, the frequency and percentage of responses were calculated. For ranking questions, responses were calculated using standard methods for weighted averages – that is, weighted average = (W1X1 + W2X2 + …)/(total number of responses), where W is the weight according to rank (with the highest rank given the highest weight, the lowest rank given the lowest weight) and X is the number of responses. Weighted averages are relative values that are used to compare responses. Open-ended questions were analysed thematically and classified by theme.

## Results

**Figure 1:**
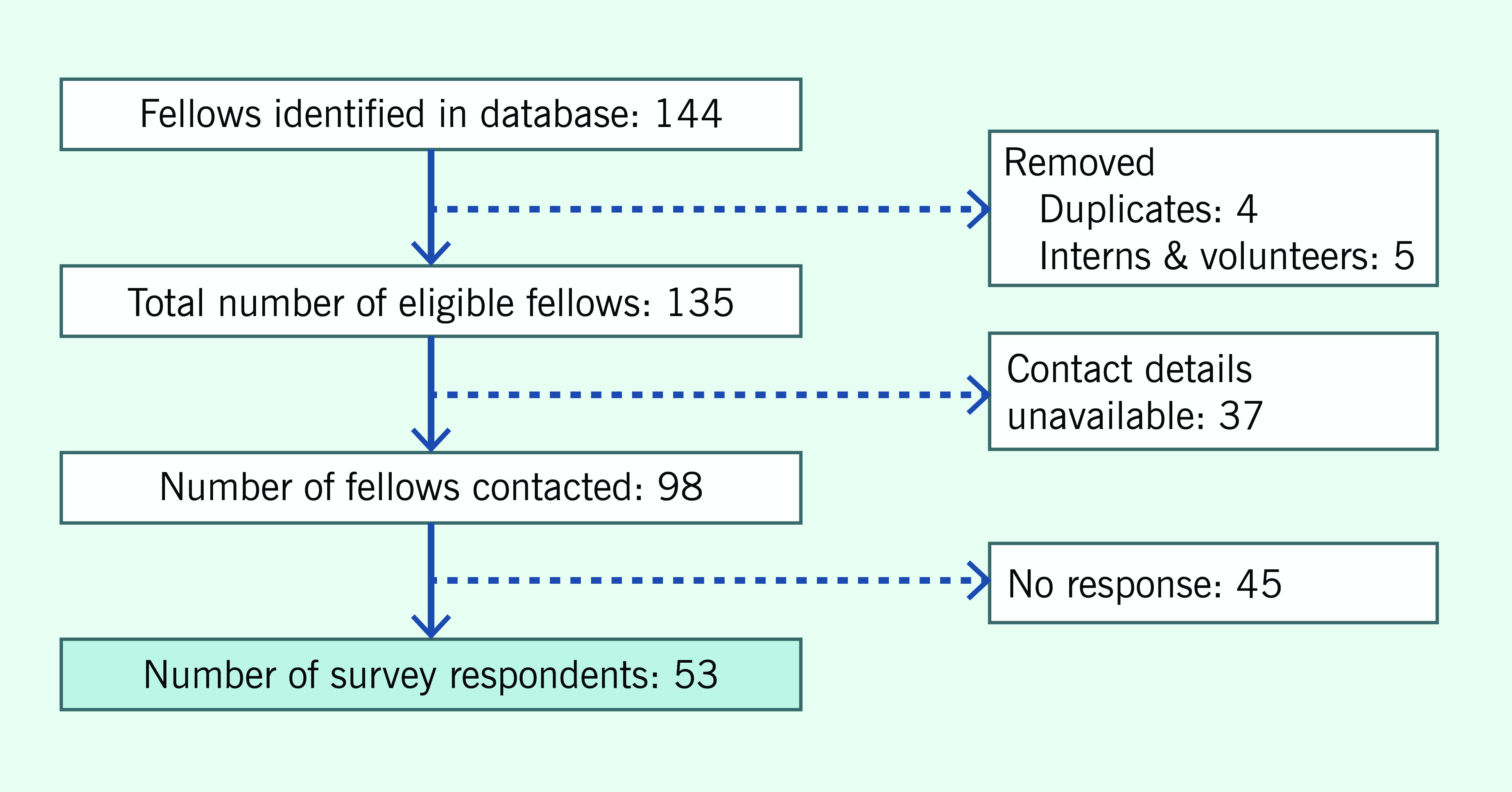
Indentification of eligible respondents for the 2018 survey of former Fellows in WHO’s Western Pacific Region Field Epidemiology Fellowship Programme

**Figure 2:**
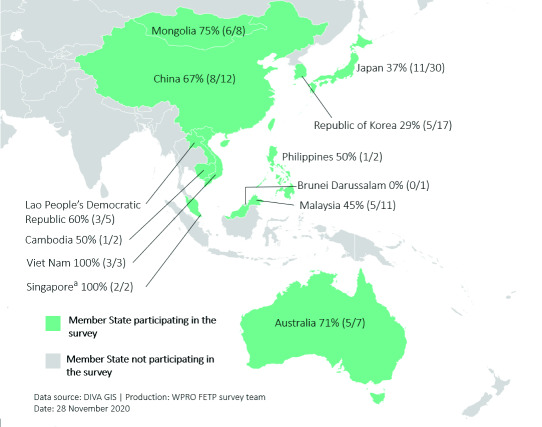
Response rate to the 2018 survey of Fellows from WHO’s Western Pacific Region Field Epidemiology Fellowship Programme, by Member State. The numerators are the number of respondents from each country; the denominators are the total number of participants in the Programme from each country during July 2006 to September 2018. (The number of respondents does not sum to 53 because three respondents did not provide information for this specific question.)

A total of 135 Regional Fellows from 12 Member States participated in the Western Pacific Region’s Field Epidemiology Fellowship Programme, of whom 20% (27/135) participated during 2006–2010, 37% (50/135) during 2011–2014 and 42% (57/135) during 2015–2018; for < 1% (1/135) the year of participation was unknown. Of these 135 Regional Fellows, 98 were contacted and 53 responded (54% response rate) from 11 countries in the Region (**Fig. 1** and **2**). Responses were received from former Regional Fellows who participated in the Programme between 2007 and 2018. Most respondents were female (62%; 33/53), affiliated with a national or subnational ministry or department of health (72%; 38/53), an FET/P graduate at the time of the attachment (53%; 28/53) and attached to the Regional Office for the Western Pacific for between 7 and 9 weeks (57%; 30/53); 77% (41/53) of respondents self-identified as having a background in epidemiology and public health (**Table 1**).

[**insert** Figure 1]

[**insert** Figure 2]

**Table 1:** Demographic information about respondents to the 2018 survey of Fellows from WHO’s Western Pacific Region Field Epidemiology Fellowship Programme

Characteristic	Number	%
**Respondents**	**53**	**100**
Female	33	62
Median age (range)	35 (26 to 48)	
**Affiliation at time of attachment**		
National ministry or department of health	29	55
Subnational health department office	9	17
University or research institute	7	13
Health care facility (clinical practice)	3	6
Other government sector, including agriculture, veterinary, environment, security	2	4
No institutional affiliation or no response	3	6
**Affiliation with FET/P^a^ at the time of attachment (multiple selections possible)**		
FET/P graduate	28	53
FET/P fellow	18	34
FET/P supervisor or mentor	6	11
FET/P programme coordinator	2	4
Other^b^ or no response	6	11
**Duration of attachment**		
4–6 weeks	10	19
7–9 weeks	30	57
10–12 weeks	9	17
> 12 weeks	1	2
No response	3	6
**Self-identified professional background (multiple selections possible)**		
Epidemiology and public health	41	77
Medicine	13	25
International development	3	6
Nursing	2	4
Veterinary medicine	2	4
Laboratory science	2	4
Pharmacology	1	2

^a^ FET/P is defined as FET, FETP or equivalent programmes that are implemented in individual countries.

^b^ “Other” included one international FET/P candidate in Thailand who was also a graduate of FET/P China, one staff at Chinese Center for Disease Control and Prevention working closely with FET/P China, and one respondent who indicated no institutional affiliation.

The majority of Fellows (66%; 35/53) indicated they thought the duration of the Programme was of an appropriate length: of these, 21 participated for 7–9 weeks, 4 participated for 4–6 weeks, 8 participated for 10–12 weeks, 1 participated for > 12 weeks and 1 participant did not provide a response for this question. Among the 23% (12/53) of participants who indicated that the duration was too short, 6 participated for 8 weeks, 4 participated for 4 weeks, 1 participated for 6 weeks and 1 participated for 10 weeks. Three Fellows (6%; 3/53) indicated that the Programme was too long, all of whom participated for 8 weeks. Three Fellows (6%, 3/53) did not provide a response for this question.

### Assessment of the Western Pacific Region’s Field Epidemiology Fellowship Programme

The skills gained during the Programme that helped Regional Fellows most in their current position were, in order of importance, (i) event-based surveillance (signal detection and screening); (ii) risk assessment; (iii) IHR (2005)-related communications and other communications, including signal verification; (iv) ability to work with a diverse group of professionals and with professionals from different countries; (v) knowledge of WHO’s role and function in health emergencies; and (vi) oral presentation skills in English.

Most respondents agreed (53%; 28/53) or somewhat agreed (32%; 17/53) that they were given clear guidance and supervision during the Programme. By year of participation, 67% (6/9) of those who participated in the individual mentorship-based Programme between 2007 and 2010 and 94% (33/35) of those who participated in the structured Programme between 2011 and 2018 agreed or somewhat agreed that they were given clear guidance and supervision during the Programme. Regional Fellows expected to gain experience and knowledge in the areas of (i) risk assessment (25%; 13/53), (ii) event-based surveillance (21%; 11/53), (iii) the structure and function of WHO in health emergencies (19%; 10/53), and (iv) outbreak and emergency responses (15%; 8/53). Most former Fellows agreed (55%; 29/53) or somewhat agreed (32%; 17/53) that their expectations of the Programme had been met. Some reasons why expectations were not considered to have been met included a lack of field deployment, having to take on a teaching role for other Fellows which impeded their own learning, and difficulty understanding the context of the risk assessment.

### Career progression and FET/P affiliation

Most former Regional Fellows were currently based in the country in which they had completed their FET/P (87%; 46/53), indicating a high retention rate in the country in which they were trained.

Their affiliations at the time of the survey in 2018 were a national ministry or department of health (55%; 29/53), subnational health department (17%; 9/53) or a university or research institute (13%; 7/53). Two former Regional Fellows worked at WHO, one as a staff member and one through an epidemiology consulting company.

Altogether, 64% (34/53) did not change their affiliation from when they joined the Western Pacific Region’s Fellowship Programme to when they participated in the survey. Among those who changed affiliations, five changed from a national or subnational health department to a university, research institute or other organization; one moved from a subnational to a national health department; one moved from a national to a subnational health department; and four moved from a university, research institute or other organization to a governmental institution.

Former Regional Fellows currently engage in surveillance and risk assessment (81%; 43/53), outbreak management (66%; 35/53), rapid response activities in their country (60%; 32/53), health emergency events (57%; 30/53) and rapid response activities outside of their country (25%; 13/53). It was reported that the Programme had helped 77% (41/53) of respondents move to a better career position with greater responsibility. Stratified by the year of participation, the Programme helped 87% (13/15) of Regional Fellows who had participated between 2007 and 2012 and 69% (20/29) of those who had participated between 2013 and 2018 in their career progression.

A majority (61%, 11/18) of those who were FET/P fellows, 64% (18/28) of those who were FET/P graduates and all (100%, 6/6) of those who were FET/P supervisors or mentors at the time they were Regional Fellows continued to be involved in FET/P programmes in various leadership roles (**Table 2**). Altogether, 3 of 11 former FET/P fellows; 5 of 18 FET/P graduates (another 5 of 18 did not indicate their years of participation); and 3 of 6 former FET/P supervisors or mentors who were affiliated with FET/Ps at the time of the survey were Regional Fellows between 2007 and 2012.

**Table 2:** Changes in the affiliations of Regional Fellows who had an association with FET/Ps from the time of participation in WHO’s Western Pacific Region Field Epidemiology Fellowship Programme to the time of the survey in 2018

Position in FET/P at the time of Regional Fellowship^a^		Affiliated with FET/P at the time of the 2018 survey^b^				
-	*n*	In any capacity	FET/P supervisor	FET/P teacher, trainer, lecturer	FET/P programme coordinator or facilitator	Host for overseas FET/P fellows
FET/P fellow	18	11	5	5	3	N/A
FET/P graduate	28	18	11	10	2	1
FET/P supervisor or mentor	6	6	5	2	3	N/A

Almost all respondents were available and willing to take part in response activities to address outbreaks or public health emergencies within (98%; 52/53) or outside of (79%; 42/53) the country in which they were based. Only 40% (21/53) had engaged in such response activities outside of their country since completing the Regional Fellowship at the time of the survey. All but one alumni (98%; 52/53) indicated that they have epidemiological expertise in public health emergencies, and 53% (28/53) have expertise in infection prevention and control.

### Feedback and recommendations

The top three aspects that the Regional Fellows most liked about the Programme were (i) working in a diverse team with a good professional support system; (ii) gaining insight into the WHO system and response systems in other countries; and (iii) learning about surveillance – that is, about collecting information, and verifying, analysing and managing it, and conducting risk assessments.

When Regional Fellows were asked about the shortcomings of the Programme, the top three responses were (i) the need for extended working hours at times; (ii) the need to start tasks early in the day; and (iii) challenges with the team structure and mentoring. When asked which technical aspects could be improved, the top three concerns were (i) the lack of, or limited, time allocated for field work in countries; (ii) the limited number of analytical or in-depth projects; and (iii) limited learning or discussion sessions. Respondents suggested providing more opportunities for field work or field investigation (*n* = 5), developing a structure to allow for continued collaboration and networking among Regional Fellows after completion of the Programme (*n* = 5), reducing the workload of WHO staff to ensure they have more time for mentoring (*n* = 3) and providing more opportunities for in-depth projects, such as analytical tasks and programme assessments (*n* = 3). Other suggestions included enrolling more participants from developing countries; ensuring that information about the objectives, setting and scope of the Programme are shared with potential Fellows before them enrolling; and providing Regional Fellows with opportunities to interact with other teams and divisions at the Regional Office for the Western Pacific.

Almost all former Regional Fellows (96%; 51/53) wished to stay in contact with the Fellowship Programme and other former Fellows. Overall, 94% (50/53) of former Fellows would either highly recommend or recommend the Programme to their colleagues.

## Discussion

The Western Pacific Region’s Field Epidemiology Fellowship Programme is unique within WHO and is designed to build capacity for detecting and responding to emerging infectious diseases and other acute public health events in the Region, in keeping with the objectives of APSED III. (*1*) Our findings provide insights into the experience of the Regional Fellows who have completed the Programme. We found that these experiences were positive and that Regional Fellows felt they had gained new skills and knowledge that have enabled them to progress in their careers. Alumni of the Regional Fellowship Programme perform key health security roles, particularly within governmental systems, and directly contribute to managing health emergencies within their countries, in the Region and globally.

Individuals who participated in the Programme continue to be involved in national FET/Ps in their home countries in supervisory, coordinating or teaching roles. The guidance from alumni in leadership roles who have gained technical and interpersonal skills through the Programme plays a key role in providing good mentorship to trainees and implementing a successful FET/P. (*7*, *8*) Through mentoring, teaching, training, supervising and directly working with FET/Ps, there are opportunities for competencies gained through the Regional Fellowship to be passed down to the next generation of trainees, which could further contribute to strengthening countries’ capacities to address health emergencies.

The Regional Fellowship Programme is helping to maintain connections and communication among Member States, and between Member States and WHO, because it is uniquely designed to bring together professionals from a variety of disciplines, nations and experiences. This diversity enriches the Regional Fellows’ experiences through mutual learning and cross-cultural interaction, and it helps them gain competencies to respond to health emergencies in various contexts. (*9*) Former Fellows ranked the team’s diversity as the most important characteristic of the Programme, and almost all Fellows wished to stay in contact with the Programme and other Fellows through a more structured channel, in addition to personal communications. The Regional Office for the Western Pacific responded to this feedback and brought Fellowship alumni together for the first alumni meeting in Tokyo, Japan, in November 2018 to continue fostering robust and long-term relationships among alumni in the Region. (*10*)

In combination with official platforms and communication channels, this alumni network could act as an incubator for catalysing new ideas and implementing innovative tools in the Region. For example, the network could play a key role in familiarizing public health officials with and supporting implementation of useful tools such as epidemic analysis for response decision-making, which aims to utilize multisource data for decision-making during an emergency response, (*11*) thereby facilitating timely detection and rapid responses.

The Western Pacific Region’s Field Epidemiology Fellowship Programme has trained a pool of experts who can be recruited to respond to health emergencies, as evidenced by the response to the novel coronavirus disease 2019 pandemic. From January to October 2020, seven former graduates contributed to the pandemic response as part of WHO’s Incident Management Support Team at the Regional Office and at WHO headquarters, according to an internal tally; many more alumni are contributing to the response through their respective governments. (*12*) The Regional Fellowship is fulfilling one of its objectives by building a workforce to provide surge capacity for public health emergencies in the Region.

Alumni highlighted a desire to gain field experience and the need for opportunities for more in-depth analytical and project-based work. In this regard, the Regional Fellows’ training experiences could be augmented by work with national FET/Ps, such as through field investigations and epidemiological analyses. Additionally, in response to suggestions from this survey, the Regional Office has modified the daily team schedule to allow Regional Fellows to complete tasks without working extended hours. Sharing information about the objectives, setting and scope of the Programme before participants apply to and participate in it is key to setting expectations for incoming Fellows.

There are limitations to this survey, such as the relatively low response rate and small sample size. Nevertheless, it is the first study to comprehensively summarize the outcomes of the Regional Fellowship Programme. The findings of this survey have been and will be used to continually improve the Programme.

Within the APSED III framework, the Regional Fellowship Programme is effective for training future leaders in field epidemiology to respond to health emergencies, developing professional relationships among Member States in the Region, and strengthening national and regional capacities. The Regional Fellowship model may be applicable to similar settings.
